# MRNIP limits ssDNA gaps during replication stress

**DOI:** 10.1093/nar/gkae546

**Published:** 2024-06-25

**Authors:** Laura G Bennett, Ellen G Vernon, Vithursha Thanendran, Caryl M Jones, Amelia Gamble, Christopher J Staples

**Affiliations:** North West Cancer Research Institute, North Wales Medical School, Bangor, Gwynedd, Wales LL57 2UW, UK; North West Cancer Research Institute, North Wales Medical School, Bangor, Gwynedd, Wales LL57 2UW, UK; North West Cancer Research Institute, North Wales Medical School, Bangor, Gwynedd, Wales LL57 2UW, UK; North West Cancer Research Institute, North Wales Medical School, Bangor, Gwynedd, Wales LL57 2UW, UK; North West Cancer Research Institute, North Wales Medical School, Bangor, Gwynedd, Wales LL57 2UW, UK; North West Cancer Research Institute, North Wales Medical School, Bangor, Gwynedd, Wales LL57 2UW, UK

## Abstract

Replication repriming by the specialized primase-polymerase PRIMPOL ensures the continuity of DNA synthesis during replication stress. PRIMPOL activity generates residual post-replicative single-stranded nascent DNA gaps, which are linked with mutagenesis and chemosensitivity in BRCA1/2-deficient models, and which are suppressed by replication fork reversal mediated by the DNA translocases SMARCAL1 and ZRANB3. Here, we report that the MRE11 regulator MRNIP limits the prevalence of PRIMPOL and MRE11-dependent ssDNA gaps in cells in which fork reversal is perturbed either by treatment with the PARP inhibitor Olaparib, or by depletion of SMARCAL1 or ZRANB3. MRNIP-deficient cells are sensitive to PARP inhibition and accumulate PRIMPOL-dependent DNA damage, supportive of a pro-survival role for MRNIP linked to the regulation of gap prevalence. In MRNIP-deficient cells, post-replicative gap filling is driven in S-phase by UBC13-mediated template switching involving REV1 and the TLS polymerase Pol-ζ. Our findings represent the first report of modulation of post-replicative ssDNA gap dynamics by a direct MRE11 regulator.

## Graphical Abstract

**Graphical Abstract:**
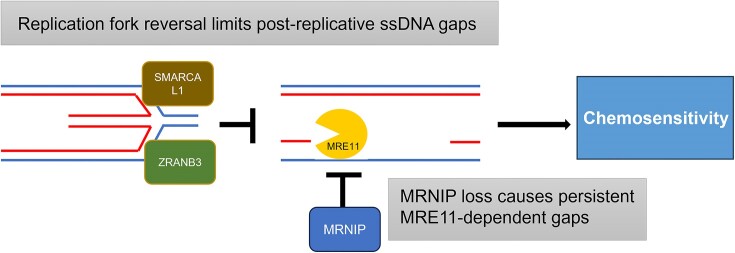

## Introduction

Genomic DNA is subject to a range of insults, from both endogenous and exogenous sources. A subset of these lesions impede the progress of the DNA replication apparatus and threaten genome integrity. Multiple mechanisms coordinate to stabilize the stalled replication fork and ensure the effective continuity of genome duplication (1,2). The global physical remodelling (reversal) of replication forks by DNA translocases including SMARCAL1 and ZRANB3 is a core element of the replication stress response (3–5). During reversal, nascent DNA rehybridizes to form a 4-way intermediate with a vulnerable DNA end, and several recognized DNA repair factors (BRCA1, BRCA2, RAD51) act collectively to protect these sites from the action of nucleases such as MRE11 and EXO1 (6,7). Our recent work demonstrates that the MRE11 interactor MRNIP represses MRE11 exonuclease activity *in vitro* and protects the regressed arm of reversed forks from MRE11-dependent degradation (8).

Many lesions are repaired by relatively slow multi-step processes, and organisms have evolved additional mechanisms to ensure the continuity of DNA replication *in lieu* of repair. Several studies elegantly demonstrate that the DNA primase-polymerase PRIMPOL facilitates replication progression via repriming in response to genotoxic stress (9–11). Repriming comes at a price, because PRIMPOL activity generates vulnerable ssDNA discontinuities in the nascent strand (Daughter Strand Gaps, DSGs), which must be filled via post-replicative gap-filling mechanisms to avoid the consequent generation of DNA breaks and genome instability (12,13). Several Translesion Synthesis (TLS) factors have been implicated in gap filling in human cells (14–16). Recent evidence suggests that fork reversal limits repriming and *vice versa* (17), and thereby the prevalence of post-replicative ssDNA gaps, as evidenced by elevated DSGs in cells deficient in fork reversal factors or treated with PARP inhibitors such as Olaparib (18,19). PARP inhibition is thought to suppress fork reversal by promoting premature RECQ1-mediated restart (20). Post-replicative gap-filling mechanisms occur via two distinct pathways in a cell cycle phase-specific manner, namely Template-Switching (TS) in S-phase, or TLS in G2^19^. Both mechanisms are dependent on the Y-family polymerase REV1 and the TLS polymerase complex Pol-ζ and are facilitated by PCNA ubiquitination (19). TLS is driven by PCNA-polymerase interaction mediated by RAD18-dependent PCNA monoubiquitination at K164. Conversely, TS-mediated post-replicative gap filling is governed by UBC13-dependent PCNA polyubiquitination.

The action of DNA repair nucleases such as MRE11 must be tightly regulated, to prevent unlicensed degradative events that threaten genome integrity. The canonical tumour suppressors BRCA1 and BRCA2 limit the prevalence of PRIMPOL and MRE11-dependent post-replicative DSGs in response to the DNA cross-linking chemotherapy cisplatin (19,21). MRE11 is a 3′-5′ exonuclease, and a recent report demonstrates that ssDNA gap processing is bidirectional and driven by both MRE11 and the 5′-3′ nuclease EXO1 (22). Post-replicative filling of DSGs in BRCA-deficient cells has been linked to the activities of both the A-family polymerase Pol-θ and the B-family polymerase Pol-ζ. (19, 21, 23,24) Despite these advances in understanding, how MRE11 is regulated in the context of its action upon ssDNA gaps remains poorly understood. Here, we employ cells deficient in the MRE11 interactor MRNIP (MRN-Interacting Protein) as a model system to further explore the dynamics and processing of DSGs. We previously characterized MRNIP as an *in vitro* repressor of MRE11 exonuclease activity that functions to protect the regressed arm of reversed forks during chronic HU treatment (8). The *in vivo* functionality of MRNIP has been recently evidenced by studies reporting impaired meiotic progression and male infertility in MRNIP KO mice – a phenotype shared with a number of other DNA repair and replication stress response factors (25,26). Here, we employ modified DNA fibre assays utilizing the ssDNA-digesting S1 nuclease to demonstrate that MRNIP has an additional role in limiting the prevalence of post-replicative DSGs. Prevention of fork reversal via transient PARP inhibition or depletion of DNA translocases leads to excessive DSGs in MRNIP KO cells. The increased prevalence of DSGs in MRNIP KO cells is dependent on PRIMPOL-mediated repriming, as well as on MRE11 exonuclease activity. MRNIP KO cells exhibit PRIMPOL-dependent DNA damage and sensitivity to the PARP inhibitor Olaparib. Furthermore, we demonstrate via a novel modified DNA fibre assay that intra-S phase post-replicative repair in MRNIP KO cells is driven largely by Pol-ζ via TS, and that small-molecule inhibition of REV1 drives DNA damage in MRNIP KO cells, particularly in the context of impaired fork reversal. In summary, this is the first report of suppression of DSGs and chemosensitivity by a direct MRE11 regulator.

## Materials and methods

### Cell culture and CRISPR-Cas9 cell line generation

WT, MRNIP KO and BRCA2 KO HeLa cells, MRNIP KO HCT116 cells, and U2OS cells stably expressing AcGFP-RPA3-RPA1-RPA2 (Super-RPA U2OS, a gift from Humphreys lab, Sheffield) were maintained as adherent monolayers in Dulbecco's Modified Eagle's medium (DMEM) with 10% Foetal Bovine Serum at 37°C in an atmosphere of 5% CO_2_. Stable MRNIP KO CRISPR clones were generated via transfection of HeLa or HCT116 cells with Santa Cruz Biotechnology CRISPR-Cas9 gRNA and HDR plasmids (sc-412131-KO-2 and sc-412131-HDR-2). The following day, the cells were reseeded into 10-cm plates in the presence of puromycin (2 μg/ml). After a further 10 days, individual clones were picked, subcultured, and analyzed by genomic PCR, qRT-PCR, and Western blotting to confirm deletion of MRNIP. The characterization of the clones used in this study has been previously reported (8).

### RNAi transfections

Cells were transfected with 10–50 nM siRNA according to cell type using Lipofectamine RNAiMAX (Invitrogen) according to the manufacturer's instructions. Cells were subsequently further treated, lysed or fixed for analysis at least 48 hrs post-transfection unless otherwise indicated.

### Cell lysis and western blotting

For the preparation of whole-cell extracts, cells were solubilized in lysis buffer [25 mM Tris–HCl (pH 7.4), 150 mM NaCl, 1% Triton X-100, 1 mM dithiothreitol (DTT), and 1 mM MgCl_2_] supplemented with Benzonase (50 U/ml) (Novagen) and cOmplete protease inhibitors and PhosSTOP phosphatase inhibitors (Roche). Lysates were clarified by centrifugation at 16 000*g* for 15 min at 4°C. Gel electrophoresis was performed using 4–12% NuPAGE gels (Invitrogen). Briefly, samples were resolved in MOPS running buffer and transferred to polyvinylidene difluoride (PVDF) membranes, which were then probed for the protein of interest using antibodies diluted in phosphate-buffered saline (PBS)–0.1% Tween 20 (Sigma-Aldrich) containing 5% Marvel or 5% BSA as appropriate. Blots were imaged on a Bio-Rad ChemiDoc machine.

### Immunofluorescence

Cells were grown on glass coverslips in 24-well trays, transfected/treated as indicated, fixed with 3% buffered paraformaldehyde for 10 min at room temperature, and permeabilized in PBS containing 0.5% Triton X-100 for 5 min at room temperature. Cells were blocked in 3% BSA and incubated with primary antibody overnight in the cold room and subsequent incubation with Alexa Fluor 488- or Alexa Fluor 594-conjugated goat anti-rabbit or anti-mouse immunoglobulin G fluorescent secondary antibodies (Invitrogen, 1:1000). Antibody dilutions and washes after incubations were performed in PBS. DNA was counterstained with 4′,6-diamidino-2-phenylindole (DAPI, 1 μg/ml), and coverslips were mounted cell-side down in Shandon Immu-Mount medium (Thermo Fisher Scientific). Fluorescence microscopy was performed on a Zeiss LSM710 confocal microscope at ×40 or ×63 magnification. Images were captured and analysed using Zen software (Zeiss).

### Native IdU assay

Cells were grown on glass coverslips in 24-well trays, transfected with relevant siRNAs, and after 24 h cells were incubated with 25 μM IdU for 24 h. Cells were then treated with genotoxins and/or inhibitors as required, and fixed in 3% buffered paraformaldehyde in PBS for 10 min at room temperature. Indirect immunofluorescence was performed using an anti-mouse BrdU antibody (BD, clone 3D4, 1:250) that cross-reacts with IdU, an Alexa anti-mouse 488 secondary antibody (Invitrogen, 1:1000) and DAPI counterstaining.

### Denaturing IdU assay

Cells were grown on glass coverslips in 24-well trays, and after 24 h cells were treated as required then incubated with 25 μM IdU for 20 min. Cells were then fixed in 3% buffered paraformaldehyde in PBS for 10 min at room temperature, and DNA denatured via a 30 min incubation with 2.5M HCl. Indirect immunofluorescence was performed using an anti-mouse BrdU antibody (BD, clone 3D4, 1:250) that cross-reacts with IdU, an Alexa anti-mouse 488 secondary antibody (Invitrogen, 1:1000) and DAPI counterstaining.

### Clonogenic survival assays

Cells were plated onto 6-well trays and transfected with siRNAs as required. Twenty-four hours later, 1000 cells were replated onto 10 cm culture dishes and treated with genotoxic agents as required after a further 24 h. Colonies were incubated for 12 days, and then were fixed and stained using methylene blue/methanol. Colonies were counted manually, and results normalized to untreated controls.

### MTT assay

Cells were plated onto 6-well trays and transfected with siRNAs as required. Twenty-four hours later, 2000 cells were replated onto 96-well culture dishes in octuplicate and treated with genotoxic agents after an additional 24 h. Cells were then incubated for 96 h, prior to the addition of 1 mg/ml Thiazolyl Blue Tetrazolium Bromide (M5655, Sigma). After 2 h, media was removed and formazan crystals dissolved in 100 μl DMSO, then absorbance at 560 nm was assessed using a spectrophotometric microplate reader. Results were normalized to untreated controls.

### DNA fibre assay

Cells were plated and transfected appropriately, then were pulse-labelled with 25 μM CldU (Sigma-Aldrich) and 250 μM IdU (Sigma-Aldrich) as indicated in combination with replication stress-inducing agents and/or inhibitors as required. Cells were then permeabilized with CSK buffer (100 mM NaCl, 10 mM MOPS, 2 mM MgCl_2_, 300 mM sucrose and 0.5% Triton X) for 5 min, then washed in S1 buffer (30 mM sodium acetate, 10 mM zinc acetate, 5% glycerol and 50 mM NaCl), then treated with 20 U/ml S1 nuclease (Thermo) in S1 buffer for 30 min. Cells were then harvested in PBS. Nuclei were then pelleted and resuspended in PBS. 2.5 μl of suspended nuclei were mixed with 7.5 μl of lysis buffer (200 mM Tris pH 7.4, 25 mM EDTA and 0.5% SDS) on a clean, dry slide (Thermo Fisher Scientific). After 7 min, slides were tilted at 30°, air-dried, then fixed in cold methanol/acetic acid (3:1). DNA fibres were denatured using 2.5 M HCl for 75 min then washed with 1× PBS before blocking in 1% Bovine Serum Albumin (BSA)/PBS containing 0.2% Tween 20 for 1 h. CldU- and IdU-labeled tracts were incubated with two anti-BrdU (5-bromo-2′-deoxyuridine) antibodies, one of which is specific for CldU (Abcam) and the other for IdU (BD). Slides were then washed and incubated with goat anti-mouse/rat Alexa Fluor 488 and Alexa Fluor 594 (Invitrogen). DNA fibres (150 per condition) were visualized on a Zeiss LSM710 confocal microscope, and images were collected using Zen software and then analyzed with ImageJ.

### List of antibodies

The following antibodies were used: Western blotting: phospho-CHK1 (133D3, Cell Signalling, 1:1000), CHK1 (Cell Signalling, 2G1D5, 1:1000), MRE11 (Abcam, ab214, 1:1000), PRIMPOL (Proteintech: 29824–1-AP, 1:1000), RAD18 (Cell Signalling, D2B8, 1:1000), UBC13 Antibody (Santa Cruz, F-10, 1:1000), REV1 Antibody (Santa Cruz, A-11, 1:1000), GAPDH Antibody (Santa Cruz, G-9, 1:2000), MRNIP Antibody (Santa Cruz, H-11, 1:500), RPA2 (Abcam 9H8, ab2175, 1:1000), Tubulin (Abcam, ab4074), SMARCAL1 (Santa Cruz Biotechnology, sc-376377), γH2AX (Cell Signaling Technology, 9718), PRIMPOL (gift from Mendez laboratory, 1:1000). Immunofluorescence: 53BP1 (Abcam, ab21083, 1:2000), IdU (BD, 3D4, 1:250) and γH2AX (Upstate, 1:1000).

### Quantitative RT-PCR

RNA was extracted using RNeasy Plus kits (Qiagen, Cat. No. 74134), and 1 μg RNA was reverse transcribed using a Superscript™ III First-strand Synthesis kit (Thermo-Fisher Cat. No. 11752050) according to the manufacturer's instructions. PCR reactions were set up in triplicate and reactions run on the Bio-Rad CFX system using the following validated Quantitect primers (GeneGlobe IDs: GAPDH (QT00079247), REV3L (QT00044653) and ZRANB3 (QT00036876), respectively) and GoTaq qPCR Master Mix (Promega Cat. No. A6001). Cycling parameters were as follows: 95°C for 2 min, then 40 cycles of melting and extension (95°C for 15 s and 60°C for 1 min). Data was collected and analysed on CFX Manager software (Bio-Rad). Triplicate Ct values for targets (REV3L, ZRANB3) were normalised to Ct values for GAPDH.

## Results

**Figure 1.**
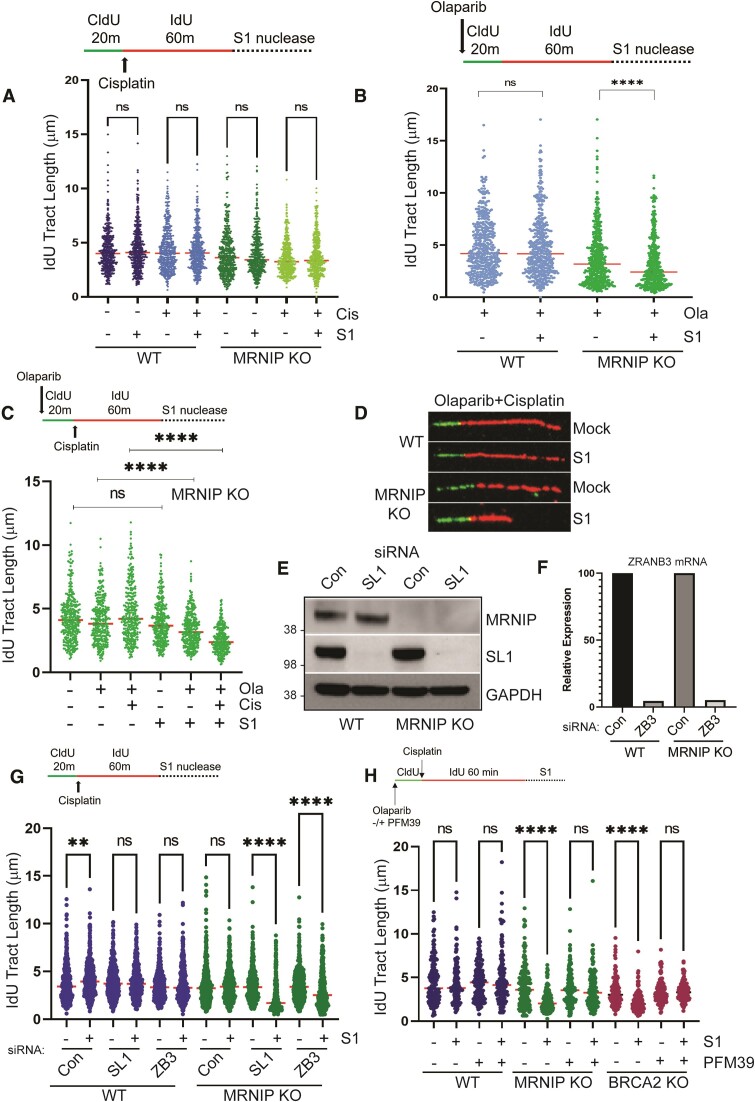
Impaired fork reversal drives MRE11-dependent post-replicative ssDNA gaps in MRNIP KO cells. (**A**) Top: DNA fibre assay schematic. WT and MRNIP KO HeLa cells were labelled with CldU for 20 min, then labelled with IdU for 60 min in the presence of PBS or 150 μM cisplatin. Cells were then treated −/+ S1 nuclease and DNA fibre assays were performed. IdU tract length was measured using ImageJ. (**B**) Top: Schematic of DNA fibre assay. WT and MRNIP KO HeLa cells were treated with 1 μM Olaparib, then nascent DNA was labelled with CldU and IdU −/+S1 nuclease. (**C**) Top: Schematic of DNA fibre assay. WT and MRNIP KO HeLa cells were treated with DMSO, 1 μM Olaparib, 150 μM cisplatin, or 1 μM Olaparib + 150 μM cisplatin, then nascent DNA was labelled with CldU and IdU −/+S1 nuclease. (**D**) Representative examples of DNA fibres from Olaparib + cisplatin-treated WT and MRNIP KO cells. (E, F) WT and MRNIP KO HeLa cells were transfected with a non-targeting control siRNA, or validated siRNAs targeting either SMARCAL1 or ZRANB3. After 48 h, cells were lysed and SMARCAL1 depletion was confirmed by Western blotting (**E**). RNA was isolated and ZRANB3 mRNA levels were quantified by qRT-PCR and normalized to GAPDH (**F**). (**G**) Top: Schematic of DNA fibre assay. WT and MRNIP KO HeLa cells were transfected with a non-targeting control siRNA, or siRNAs targeting SMARCAL1 or ZRANB3. After 48 h, nascent DNA was labelled with CldU, then IdU and cisplatin −/+ S1 nuclease. (**H**) Top: Schematic of DNA fibre assay. WT and MRNIP KO HeLa cells were treated with 1 μM Olaparib and either DMSO or 25 μM PFM39, then nascent DNA was labelled with CldU, followed by IdU labelling in the presence of 100 μM cisplatin −/+ S1 nuclease. Experiments were performed three times independently and were confirmed by at least two members of the laboratory. Results are displayed as dot plots with indicated median values and analysed via one-way ANOVA. **P< 0.01, ****P< 0.0001

Given recent reports that BRCA1 and BRCA2 restrain MRE11-dependent degradative processes at post-replicative DSGs in cisplatin-treated cells, we hypothesised that loss of the MRE11 interactor MRNIP also modulates DSGs. To interrogate this, we employed a modified DNA fibre assay incorporating the ssDNA-cleaving S1 nuclease, in previously validated MRNIP KO cells generated by CRISPR-Cas9. Ongoing sites of replication were labelled with CldU, followed by an extended IdU incubation in the presence of genotoxic challenge, then S1 treatment (see schematics in Figure 1A and throughout). Replicate wells were mock-treated to assess IdU tract length in the absence of S1 treatment, and thus account for alterations in fork progression under each condition. Decreased IdU tract length following nuclease treatment is indicative of S1 nuclease action, and by extension the presence of post-replicative ssDNA.

### Impaired fork reversal drives MRE11-dependent post-replicative ssDNA gaps in MRNIP KO cells

Treatment with the cross-linking chemotherapy cisplatin is associated with persistent post-replicative gaps in BRCA-deficient cells. Therefore, we initially performed S1 digestions following cisplatin treatment of WT and MRNIP KO HeLa cells and found that S1 treatment under these conditions did not lead to a detectable increase in DSGs (Figure 1A). Next, we assessed DSGs following treatment with the PARP inhibitor Olaparib, since PARP inhibition is reported to exacerbate ssDNA gaps in BRCA1 or BRCA2-deficient cells by limiting fork reversal (19). Loss of MRNIP in HeLa cells resulted in decreased IdU tract length following S1 treatment, indicative of a significant increase in the prevalence of DSGs following Olaparib treatment (Figure 1B).

Several lines of evidence suggest that PARP inhibition limits replication fork reversal, which in turn is reported to suppress post-replicative DSGs (3,18,19). To test whether PARP inhibition limits ssDNA gap prevalence in cisplatin-treated MRNIP KO cells, we treated these with DMSO, Olaparib, cisplatin, or a combination of Olaparib and cisplatin prior to IdU labelling and S1 treatment. Again, cisplatin treatment did not result in a significant increase in DSGs, while a significant reduction in IdU tract length post-S1 was observed in Olaparib-treated MRNIP KO cells. The prevalence of DSGs was further exacerbated in cells treated with both Olaparib and cisplatin, supportive of the hypothesis that PARP inhibition limits DSGs in cisplatin-treated cells (Figure 1C). Confocal microscopy images of representative fibres are displayed (Figure 1D). We hypothesized that MRNIP limits DSGs formed in the absence of functional fork reversal mechanisms. Since the effects of PARP inhibitors are not limited to impairment of fork reversal, we sought to further test the connection between reversal and DSGs by conducting S1 fibre assays in WT and MRNIP KO cells depleted of the fork reversal factors SMARCAL1 or ZRANB3, followed by IdU labelling in the presence of cisplatin, but in the absence of PARP inhibitor. Depletion of SMARCAL1 and ZRANB3 was confirmed in both WT and MRNIP KO cells by Western blotting and qRT-PCR, respectively (Figures 1E and F). DSGs were undetectable in WT cells following SMARCAL1 or ZRANB3 depletion, though depletion of either factor in MRNIP KO cells led to a marked increase in ssDNA gaps (Figure 1G), lending support to the concept that fork reversal limits DSGs.

The nuclease MRE11 has been implicated in modulation of DSGs in multiple models. To test whether the observed gaps in MRNIP KO cells are MRE11-dependent, we assessed DSGs in Olaparib and cisplatin-treated WT and MRNIP KO cells pre-treated with either DMSO or the MRE11 exonuclease inhibitor PFM39 (27). BRCA2 KO HeLa cells were included as a positive control. Again, the combination of PARP inhibition and cisplatin treatment did not lead to detectable ssDNA gaps in WT cells post-S1 treatment. As expected, IdU tracts were markedly shorter in BRCA2-deficient cells following S1 treatment, and a similar post-S1 reduction in IdU tract length was observed in MRNIP KO HeLa cells (Figure 1H). MRE11 inhibition via PFM39 treatment led to a complete reversal of the gap phenotype in both MRNIP and BRCA2 KO models (Figure 1H). Gaps in MRNIP KO cells were also reversed by treatment with the MRE11 exonuclease inhibitor mirin (Supplementary Figure S1B), indicating a specific role for the MRE11 exonuclease in processing of DSG sites in independent models of increased gap prevalence. We also noted that loss of MRNIP, or treatment with Olaparib and Cisplatin did not alter the proportion of replicating cells as assessed by denaturing IdU assay, or the phosphorylation of the S-phase checkpoint kinase CHK1 (Supplementary Figure S1C and D). Total MRE11 levels were unaffected by MRNIP loss, and the proportion of cells positive for MRE11 foci was unaltered in MRNIP KO cells treated with Olaparib and cisplatin (Supplementary Figure S2A and B). We did observe increased chromatin association of MRE11 in untreated MRNIP KO cells (Supplementary Figure S2C). This elevated association may be reflective of the increased DNA damage observed in cycling MRNIP KO cells.

### ssDNA gaps in MRNIP KO cells are driven by replication repriming.

**Figure 2.**
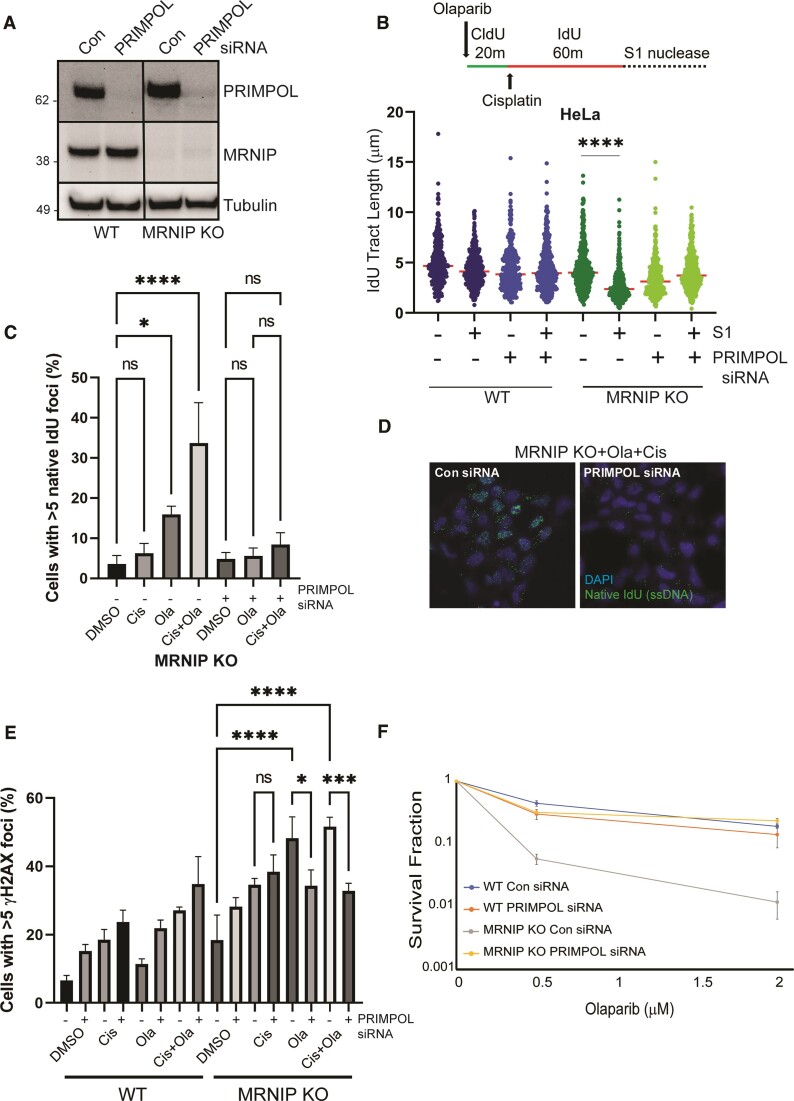
ssDNA gaps in MRNIP KO cells are driven by replication repriming. (**A**) WT and MRNIP KO HeLa cells were transfected with either a non-targeting control siRNA, or a validated siRNA targeting PRIMPOL. After 48 hrs, whole cell extracts were generated and resolved by Western blotting with the indicated antibodies. (**B**) Top: DNA fibre assay schematic. WT and MRNIP KO HeLa cells were transfected as in (A), then treated with Olaparib and cisplatin as in the indicated schematic, followed by S1 nuclease treatment. Results are displayed as dot plots with indicated median values. (**C**) WT and MRNIP KO HeLa cells were transfected with control siRNA or an siRNA targeting PRIMPOL. After 24 h, cells were incubated for 16 h with 25 μM IdU, then treated with 100 μM cisplatin or 1 μM Olaparib alone or in combination for 4 h. Cells were fixed and stained with a BrdU antibody that cross-reacts with IdU. IdU-positive cells were counted, and results displayed as a proportion of the total number of cells. (**D**) Representative images of native IdU staining in Olaparib and cisplatin-treated MRNIP KO cells, following transfection with control siRNA or PRIMPOL siRNA. (**E**) WT and MRNIP KO HeLa cells were transfected with a non-targeting control siRNA, or an siRNA targeting PRIMPOL. Forty-eight hours later, cells were treated with either DMSO, 100 μM cisplatin, 1 μM Olaparib, or both drugs for 16 h, then were fixed and stained with a γH2AX antibody, and counterstained with DAPI. Cells with more than 5 foci were counted and results plotted as a percentage of the total number of cells. (**F**) WT and MRNIP KO cells were transfected as in (A). After 48 h, cells were replated and treated with the indicated concentrations of Olaparib. After 12 days, surviving colonies were counted and results normalised to untreated controls. All experiments were performed three times independently and analysed using one-way ANOVA. *P< 0.05, ***P< 0.001, ****P< 0.0001

Post-replicative ssDNA gaps in BRCA1/2-deficient cells form consequent to PRIMPOL-mediated repriming (19). To test whether the gaps observed in MRNIP KO cells are generated by repriming, we depleted PRIMPOL in WT and MRNIP KO HeLa cells and treated these with Olaparib and cisplatin. PRIMPOL depletion was confirmed by Western blotting (Figure 2A). S1 nuclease-linked DNA fibre assays revealed that the increase in gaps observed in MRNIP KO cells is reversed by PRIMPOL depletion (Figure 2B), indicating that repriming drives the formation of ssDNA gaps in MRNIP KO cells. This finding was also confirmed in MRNIP KO HCT116 cells (Supplementary Figure S1E and F). To validate these phenotypes further, we performed native IdU assays, which facilitate visualization of ssDNA-containing regions. In agreement with our DNA fibre analysis, treatment with cisplatin did not lead to a significant increase in ssDNA in MRNIP KO cells. Conversely, a moderate increase in ssDNA formation was observed in MRNIP KO cells following Olaparib treatment, and this was exacerbated further by pre-treatment with Olaparib prior to cisplatin exposure. Furthermore, the observed increase in ssDNA formation was completely reversed by depletion of PRIMPOL (Figure 2C and D).

The increased prevalence of ssDNA gaps has been linked to elevated DNA damage and chemosensitivity (18,19,21,23,28). Given that we observe increased gaps in MRNIP KO cells following single-agent Olaparib treatment, or combinatorial treatment with both Olaparib and cisplatin, we set out to assess the consequent effects on DNA damage levels and cell survival in response to these agents, via clonogenic survival assays, and the detection of γH2AX. As published previously (8), we found that MRNIP KO cells exhibited an increase in basal DNA damage markers in cycling cells, though we also observed additional increases in both damage markers following treatment with Olaparib and cisplatin, either alone or in combination. Increased DNA damage in MRNIP KO cells was partially ameliorated by depletion of PRIMPOL, suggesting a causative role for replication repriming (Figure 2E). MRNIP KO HeLa cells also exhibited PRIMPOL-dependent Olaparib sensitivity in clonogenic assays (Figure 2F). Relative to WT cells, MRNIP KO HeLa cells also displayed PRIMPOL-dependent sensitivity to increasing doses of cisplatin at a fixed dose of Olaparib (Supplementary Figure S1A). This latter finding correlates with the enhanced prevalence of DSGs in MRNIP KO cells treated with both Olaparib and Cisplatin.

### Fork reversal and REV1 limit ssDNA gaps and DNA damage markers in MRNIP KO cells

**Figure 3.**
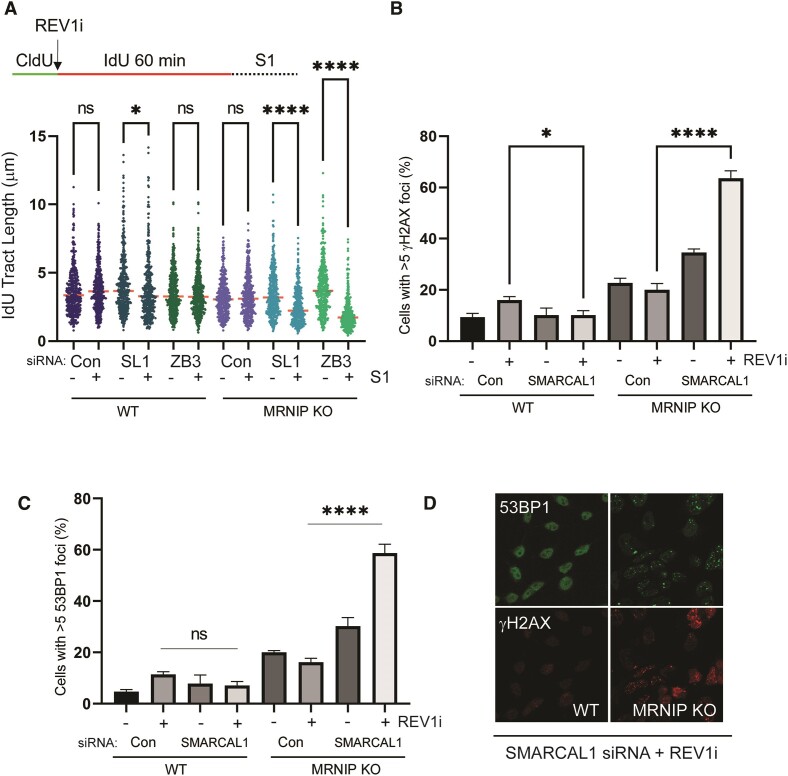
Fork reversal and REV1 limit ssDNA gaps and DNA damage markers in MRNIP KO cells. (**A**) Top: Schematic of DNA fibre assay. WT and MRNIP KO HeLa cells were transfected with a non-targeting control siRNA, or siRNAs targeting either SMARCAL1 or ZRANB3. After 48 h, cells were treated with 1 μM JH-RE-06 (REV1 inhibitor), then nascent DNA was labelled with CldU for 20 min, followed by IdU for 60 min. ssDNA was then digested with S1 nuclease. IdU tract length was measured using ImageJ. IdU tract length was measured using ImageJ. (B–D) WT and MRNIP KO cells were transfected with a control siRNA, or an siRNA targeting SMARCAL1. Forty-eight hours later, cells were treated with either DMSO or 1 μM JH-RE-06 for 16 hrs, then fixed and stained with antibodies against γH2AX (**B**) and 53BP1 (**C**), and counterstained with DAPI. Cells with more than 5 foci were counted and results plotted as a percentage of the total number of cells. Representative images are displayed in (**D**). All experiments were performed three times independently and analysed via one-way ANOVA. *P< 0.05, ****P< 0.0001.

Given that the TLS polymerase complex POL-ζ has previously been implicated in gap-filling in BRCA1/2-deficient cells, we also assessed DSGs in MRNIP KO cells following treatment with the small molecule inhibitor JH-RE-06, which inhibits REV1/POL-ζ-dependent synthesis by enhancing REV1 dimerization (29). REV1 acts as a platform to coordinate TLS activities by binding PCNA via its N-terminal BRCT domain (30,31). Treatment with JH-RE-06 leads to increased gap prevalence in BRCA1-mutant cancer cells (21). To test the involvement of REV1 in the regulation of DSGs in MRNIP KO cells, we employed S1-linked fibre assays in WT and MRNIP KO cells treated with JH-RE-06, following depletion of the fork reversal factors SMARCAL1 or ZRANB3. While a moderate increase in ssDNA gaps was observed in JH-RE-06-treated WT cells depleted of SMARCAL1, loss of MRNIP led to a marked increase in gap prevalence following either SMARCAL1 or ZRANB3 depletion (Figure 3A). We also stained WT and MRNIP KO cells for the DNA damage markers 53BP1 and γH2AX, following depletion of SMARCAL1 and JH-RE-06 treatment. Neither SMARCAL1 depletion or JH-RE-06 treatment (alone or in combination) significantly increased DNA damage markers in WT cells. However, depletion of SMARCAL1 led to an increase in the proportion of MRNIP KO cells positive for both γH2AX and 53BP1, and this increase was further exacerbated by the addition of JH-RE-06 (Figures 3B–D). This data suggests that impaired fork reversal leads to increased DSGs and DNA damage markers in the absence of MRNIP, and that this damage is limited by the action of Pol-ζ components.

### Post-replicative gap filling in MRNIP KO cells is mediated by UBC13 and REV1/pol-ζ

**Figure 4.**
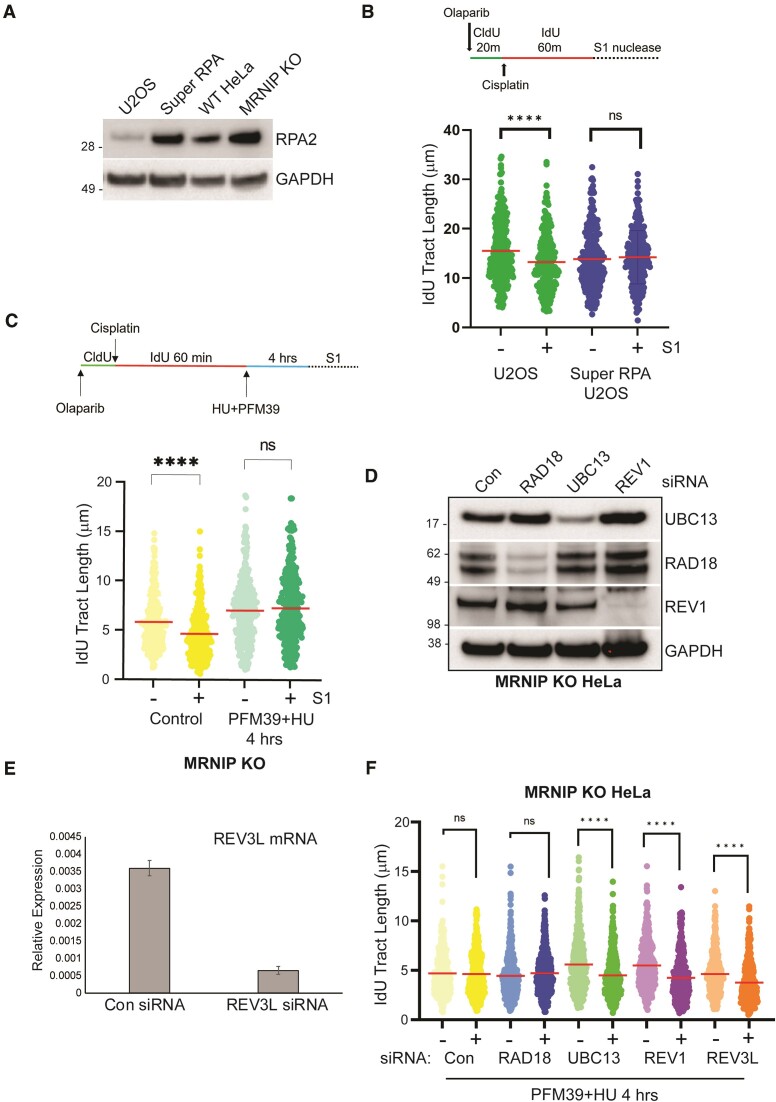
Post-replicative gap filling in MRNIP KO cells is mediated by UBC13 and REV1/Pol-ζ. (**A**) Parental U2OS cells, or Super RPA U2OS cells were lysed, and extracts were resolved by SDS-PAGE and probed via Western blotting with the indicated antibodies. (**B**) Top: Schematic of DNA fibre assay. Parental and Super RPA U2OS were treated with 1 μM Olaparib, then nascent DNA was labelled with CldU for 20 min, followed by labelling with IdU in the presence of 150 μM cisplatin for 60 min. ssDNA was then digested with S1 nuclease. IdU tract length was measured using ImageJ. (**C**) WT and MRNIP KO HeLa cells were treated with 1 μM Olaparib, then nascent DNA was labelled with CldU, followed by IdU labelling in the presence of 150 μM cisplatin to allow post-replicative gaps to form. Cells were then immediately lysed and DNA fibres spread (Control), or cells were treated with a combination of 4 mM HU and 25 μM PFM39 for 4 h, then mock-treated or S1 nuclease-treated prior to spreading. DNA fibre spreading and analysis was performed and the length of IdU tracts was measured using ImageJ. (D, E) MRNIP KO cells were transfected with the indicated siRNAs, and knockdowns were confirmed by Western blotting (**D**) or qRT-PCR (**E**). (**F**) MRNIP KO cells were transfected with the indicated siRNAs and then labelled and treated with PFM39 and HU as in (C) prior to S1 nuclease treatment and DNA fibre spreading and analysis. All experiments were performed three times independently. Data in (F) was confirmed independently by two laboratory members. Results are displayed as dot plots with indicated median values and analysed using one-way ANOVA. ****P< 0.0001.

There are differences between our findings and data already published by independent research groups using other cell lines. Modulation of fork reversal via PARP inhibition is reported to result in increased prevalence of ssDNA gaps in cisplatin-treated WT U2OS cells (19). In contrast, we did not observe detectable gaps in WT HeLa cells treated with both cisplatin and Olaparib. U2OS cells contain low levels of the ssDNA-binding protein Replication Protein A (RPA) and are thus prone to RPA exhaustion during replicative stress (32). To test whether ssDNA gap formation following Olaparib and cisplatin treatment of U2OS cells is a consequence of limited RPA levels, we employed ‘Super RPA’ U2OS cells, which express elevated physiological levels of all three RPA subunits (32). Firstly, we confirmed that U2OS cells contain markedly less RPA2 than the WT or MRNIP KO HeLa cells employed in this study, and that ‘Super RPA’ U2OS cells express an elevated level of RPA2 (approx. 2-fold higher than U2OS) that is comparable to the HeLa lines tested (Figure 4A and Supplementary Figure S2D). Secondly, we confirmed via S1-linked DNA fibre assays that co-treatment with Olaparib and cisplatin result in detectable ssDNA gaps in WT U2OS cells. Interestingly, these ssDNA gaps were suppressed in Super RPA U2OS (Figure 4B), suggesting that the apparent discrepancy between our findings and studies conducted in U2OS cells is likely due to cell line-specific differences in RPA levels.

Multiple studies have implicated the TLS polymerase complex Pol-ζ in post-replicative ssDNA gap closure, via both TS behind the replication fork in S-phase, and TLS in G2. To assess gap closure, we designed a modified DNA fibre assay in which CldU and IdU labelling in the presence of Olaparib and cisplatin was followed by an extended 4-hr HU treatment with the dual aims of prevention of further replisome progression, and the affordance of time to allow for polymerase-mediated gap closure. Post-IdU labelling, MRNIP KO cells were also treated with the MRE11 inhibitor PFM39 to prevent MRE11-dependent degradation of the reversed fork formed at the terminus of the IdU label upon HU treatment (Figure 4C, schematic). Addition of PFM39 post-labelling does not interfere with initial MRE11-dependent ssDNA gap processing since these gaps are formed by repriming and processed by MRE11 while the IdU label is present. Indeed, treatment of cells with HU and PFM39 resulted in complete abolition of the gap phenotype, indicative of polymerase-mediated gap closure during the window of HU treatment (Figure 4C). To ascertain whether POL-ζ plays a role in gap closure in MRNIP KO cells, we depleted cells of REV1 or the POL-ζ catalytic subunit REV3L and carried out the modified HU/PFM39 protocol. To determine whether this gap filling involves TS or TLS, we depleted cells of UBC13 or RAD18. Depletion of UBC13, REV1 and REV3L were confirmed by Western blotting, and REV3L mRNA depletion was confirmed by RT-qPCR, since a working antibody was not available (Figures 4D and E). As before, we did not detect ssDNA gaps in MRNIP KO cells treated with Olaparib and cisplatin following the modified gap-filling protocol, though significant ssDNA gaps were restored upon depletion of UBC13, REV1 or REV3L, but not RAD18 (Figure 4F). These findings suggest that in MRNIP KO cells, ssDNA gaps are filled in S-phase by POL-ζ via UBC13-driven TS, but not by RAD18-dependent TLS (Figure 4F). This agrees with previous work carried out by the Vindigni laboratory, which suggested that TS that the major gap-filling mechanism at play in S-phase cells (19).

In summary, our findings demonstrate that MRNIP limits post-replicative MRE11-dependent ssDNA gaps formed by replication repriming, and that gaps formed in MRNIP-deficient cells are filled by the action of REV1 and the TLS polymerase POL-ζ. This is the first report of post-replicative DSG suppression by a nuclease regulatory factor.

## Discussion

Our data builds on prior studies demonstrating that replication repriming by PRIMPOL leads to post-replicative ssDNA gaps, which must be filled in a timely manner to prevent the accumulation of genome instability (18,19). In WT cells, gaps are effectively suppressed or filled, although notably ssDNA gaps are reported to persist in BRCA1/2-deficient cells treated with replication stress-inducing agents, including traditional genotoxic chemotherapies like cisplatin and precision medicines such as PARP inhibitors. Here, we confirm that BRCA2-deficient HeLa cells display persistent post-replicative gaps upon treatment with Olaparib and cisplatin (Figure 1).

BRCA1 and BRCA2 also act to suppress MRE11-dependent degradation of HU-stalled replication forks (6). In all reports to date, post-replicative gaps observed in BRCA1/2-deficient cells are also dependent on MRE11 exonuclease activity, suggesting that similar regulatory mechanisms may act to limit nucleolytic degradation at both reversed forks and at ssDNA gaps. Such unlicensed nucleolytic gap processing prevents timely gap closure by TLS polymerases, resulting in persistence of under-replicated DNA (33). We assessed the prevalence of post-replicative gaps in cancer cells deficient in MRNIP, an MRE11 interactor that suppresses MRE11 exonuclease activity *in vitro*, and which prevents MRE11-dependent degradation of nascent DNA at stalled, reversed replication forks (8). Persistent post-replicative gaps were detected in MRNIP KO cells treated with Olaparib but not cisplatin, although Olaparib pre-treatment led to an additional elevation in gap prevalence upon cisplatin treatment. This is consistent with prior reports that Olaparib limits fork reversal in response to cisplatin (3), and promotes repriming. That gaps were not detected in MRNIP KO cells in the presence of cisplatin alone suggests clear mechanistic differences between BRCA1/2-mediated gap protection, and the protection afforded by MRNIP. This is perhaps unsurprising given that MRNIP represses MRE11 exonuclease activity, likely via direct interaction, while BRCA2 functions by facilitating RAD51 nucleation at ssDNA gaps (34) (although one report also suggests that BRCA2 limits repriming) (35). We observed increased chromatin association of MRE11 in untreated MRNIP KO cells (Supplementary Figure S2B and C). This may simply reflect the elevated DNA damage present in cycling MRNIP KO cells, although it is presently unclear if this elevated association is of direct relevance to modulation of the gap phenotype.

In further support of the hypothesis that fork reversal limits repriming during replication stress, we also observed elevated prevalence of ssDNA gaps in cisplatin-treated MRNIP KO cells depleted of the DNA translocases SMARCAL1 and ZRANB3. Additionally, the ssDNA gaps and elevated markers of DNA damage observed in MRNIP KO cells treated with both Olaparib and cisplatin are dependent on the repriming factor PRIMPOL (Figure 2). Given that PRIMPOL has evolved to promote replication during stress, it is likely that PRIMPOL-induced gaps become problematic only in the absence of regulatory factors such as BRCA1/2 and MRNIP. However, whether post-replicative ssDNA gaps always correlate with chemosensitivity remains an open question. We note that a recent report demonstrates that loss of BRCA2 fork protective and gap suppressive functions do not cause replication stress in human mammary cells (36). Conversely, work from the Zou laboratory shows that APOBEC3A expression leads to PRIMPOL-dependent ssDNA gaps, which are linked to chemosensitivity (38). Our work demonstrates that the enhanced chemosensitivity of MRNIP KO cells is PRIMPOL-dependent, though one must concede the possibility that a cryptic function of PRIMPOL may be responsible for the modulation of cellular sensitivity following genotoxic stress.

The B-family TLS polymerase REV3L is the catalytic component of the multi-subunit complex Pol-ζ. Both REV3L and the TLS master regulator REV1L have recently been implicated in post-replicative gap filling in BRCA-deficient cells (19,21). To test the potential role of these factors in MRNIP KO cells, we employed a modified nascent DNA labelling protocol to assess ssDNA gap filling in S-phase. Cells were co-treated with Olaparib and cisplatin during CldU and IdU labelling. The resultant labelled nascent strands contain ssDNA gaps formed consequent to repriming. High-dose HU was used to chronically stall the replication fork at the end of the IdU label, preventing further progression. Given that HU treatment results in rapid global fork reversal (and leads to nascent strand degradation and IdU tract shortening in MRNIP KO cells), we also treated cells with the MRE11 exonuclease inhibitor PFM39 following IdU labelling. This step prevents unwanted nascent strand degradation without interference with initial ssDNA gap processing, given that gaps are formed during IdU labelling, prior to PFM39 addition. Cells were incubated in PFM39/HU-containing media for 4 h to allow gap filling to occur. In MRNIP KO cells, this protocol resulted in a complete reversal of the ssDNA gap phenotype. Gap-filling in this model was dependent on UBC13, REV1L and REV3L. Prior evidence indicates that POL-ζ conducts extensive synthesis downstream of UV-induced damage sites in yeast, supporting the hypothesis that gaps of significant size can be successfully filled by REV3L and its partners (37). In BRCA-deficient cells, gap filling mechanisms involving Pol-ζ are also thought to be cell cycle phase-dependent. In S-phase, gap filling is driven by UBC13-dependent Template Switching, while in G2 phase filling is dependent on RAD18-driven TLS (19). Consistent with this model, S-phase gap-filling in MRNIP KO cells was unaffected by depletion of RAD18 but was dependent on UBC13. That gaps are successfully filled under these conditions tallies with the prior observation that the activity of TLS polymerases such as POL-ζ are unaffected by hydroxyurea-mediated suppression of dNTP synthesis (39). This resistance to HU treatment is likely due to the relatively low processivity of TLS polymerases, and their consequent tolerance of dNTP pool imbalances relative to the core replicative polymerases, which must coordinate rapid and continuous leading and lagging strand synthesis.

These findings also demonstrate the importance of the POL-ζ complex in mediating gap-filling outside the context of BRCA1/2-deficiency. That gap-filling can occur in MRNIP KO cells within the extended timescale of this gap-filling assay could be interpreted as being indicative of the presence of delayed, rather than defective gap-filling in MRNIP KO cells. However, since the MRE11 exonuclease inhibitor PFM39 was included in the assay to avoid the degradation of the terminal reversed fork by MRE11 in the absence of MRNIP, one must also consider the possibility that persistent post-replicative MRE11 activity is required to maintain gaps in MRNIP KO cells. This latter hypothesis is consistent with a model in which dysregulated MRE11 activity in MRNIP KO cells drives aberrant gap extension. Notably, we detected increased gap prevalence and markers of DNA damage following REV1 inhibition in MRNIP KO cells depleted of fork reversal factors. That these events occur in the absence of an exogenous genotoxin is of interest. Prior reports suggest a role for Pol-ζ in facilitating replication of difficult-to-replicate regions of the genome (40,41). Furthermore, fork reversal has been demonstrated to be a frequent occurrence during the unperturbed S-phase (42). We therefore suggest that fork reversal limits repriming at such sites, and that treatment of MRNIP KO cells with a REV1 inhibitor reveals gaps by further tipping the balance in the tug-of-war between gap-filling mechanisms and end-degradative processes.

Nucleases can act in a destructive manner if not properly regulated, though we know relatively little about the mechanisms that temper MRE11 activity. Our prior work demonstrates that MRNIP represses MRE11 exonuclease activity *in vitro*, and that loss of MRNIP results in MRE11-dependent degradation of nascent DNA at hydroxyurea-stalled forks. Here, we have extended our study of MRNIP to define its role in the regulation of post-replicative ssDNA gap processing. We demonstrate that MRNIP loss results in an increased prevalence of post-replicative ssDNA gaps, which are formed during repriming, and are driven by MRE11 activity. These gaps are limited by replication fork reversal and are filled in during S-phase via a mechanism dependent on UBC13- and the TLS polymerase complex Pol-ζ.

## Supplementary Material

gkae546_Supplemental_File

## Data Availability

All data needed to evaluate the conclusions in the paper are present in the paper. Additional data related to this paper may be requested from the authors.
