# Supplementary material for: MRNIP limits ssDNA gaps during replication stress
Source: Nucleic Acids Res. 2024 Jun 25;52(14):8320–31. doi: 10.1093/nar/gkae546 (PMC11317133; doi:10.1093/nar/gkae546)
Supplement: gkae546_Supplemental_File [file gkae546_supplemental_file.pdf]

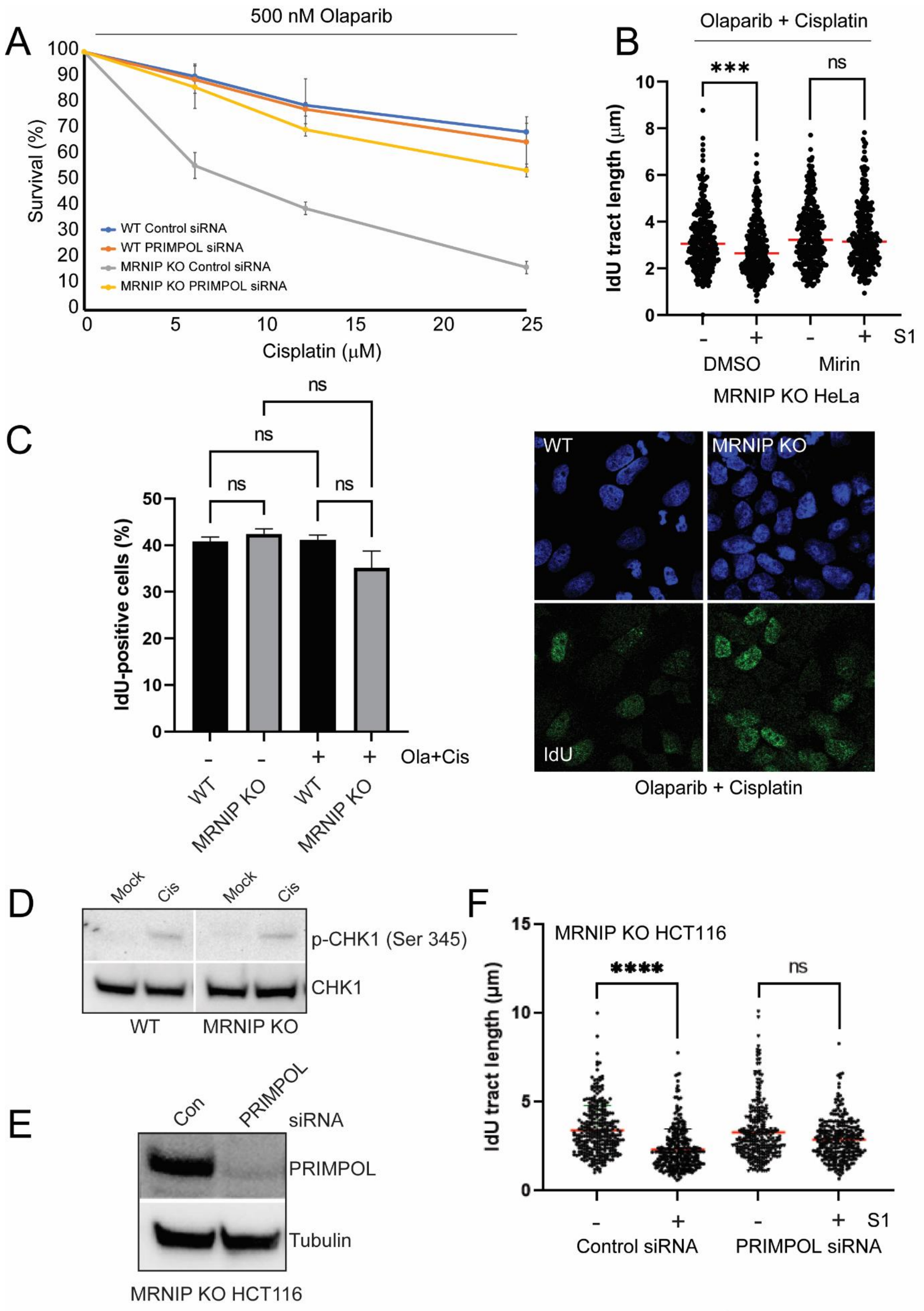

Bennett *et al.*, 2024 Supplementary Figure 1

Supplementary Figure 1: MRE11-dependent ssDNA gaps lead to PRIMPOL-driven chemosensitivity in MRNIP KO cells. **A:** WT and MRNIP KO cells were transfected with a non-targeting control siRNA, or an siRNA targeting PRIMPOL. After 24 hrs, cells were replated. Cells were then treated with 500 nM Olaparib in combination with increasing concentrations of cisplatin, as indicated. After 4 days, an MTT assay was performed, and the findings normalised to data obtained following exposure to Olaparib alone. **B:** WT and MRNIP KO HeLa cells were labelled with CldU for 20 min, followed by 1 hr IdU labelling in the presence of Olaparib and cisplatin -/+ DMSO or Mirin (25 μM), followed by S1 nuclease treatment. Results are displayed as dot plots with indicated median values. **C:** WT and MRNIP KO cells were treated with DMSO or Olaparib (1 μM) and Cisplatin (150 μM) and 1 hr later nascent DNA was labelled via a 20 min incubation with IdU. DNA was denatured using 2.5M HCl, and nascent DNA detected via indirect immunofluorescence with an anti-BrdU antibody that cross-reacts with IdU. The proportion of IdU-positive cells was quantified. **D:** WT and MRNIP KO HeLa cells were treated with 150 μM Cisplatin for 2 hrs, then whole cell extracts were prepared, and assessed via Western blotting using the indicated antibodies. **E and F:** MRNIP KO HCT116 cells were transfected with a non-targeting siRNA or an siRNA targeting PRIMPOL. PRIMPOL depletion was confirmed by Western blotting (E). After 48 hrs, cells were labelled with CldU for 20 min, followed by 1 hr IdU labelling in the presence of Olaparib and cisplatin, followed by S1 nuclease treatment. Results are displayed as dot plots with indicated median values (F). All experiments were performed three times independently and analysed using via one-way ANOVA. ***$p<0.001$, ****$p<0.0001$

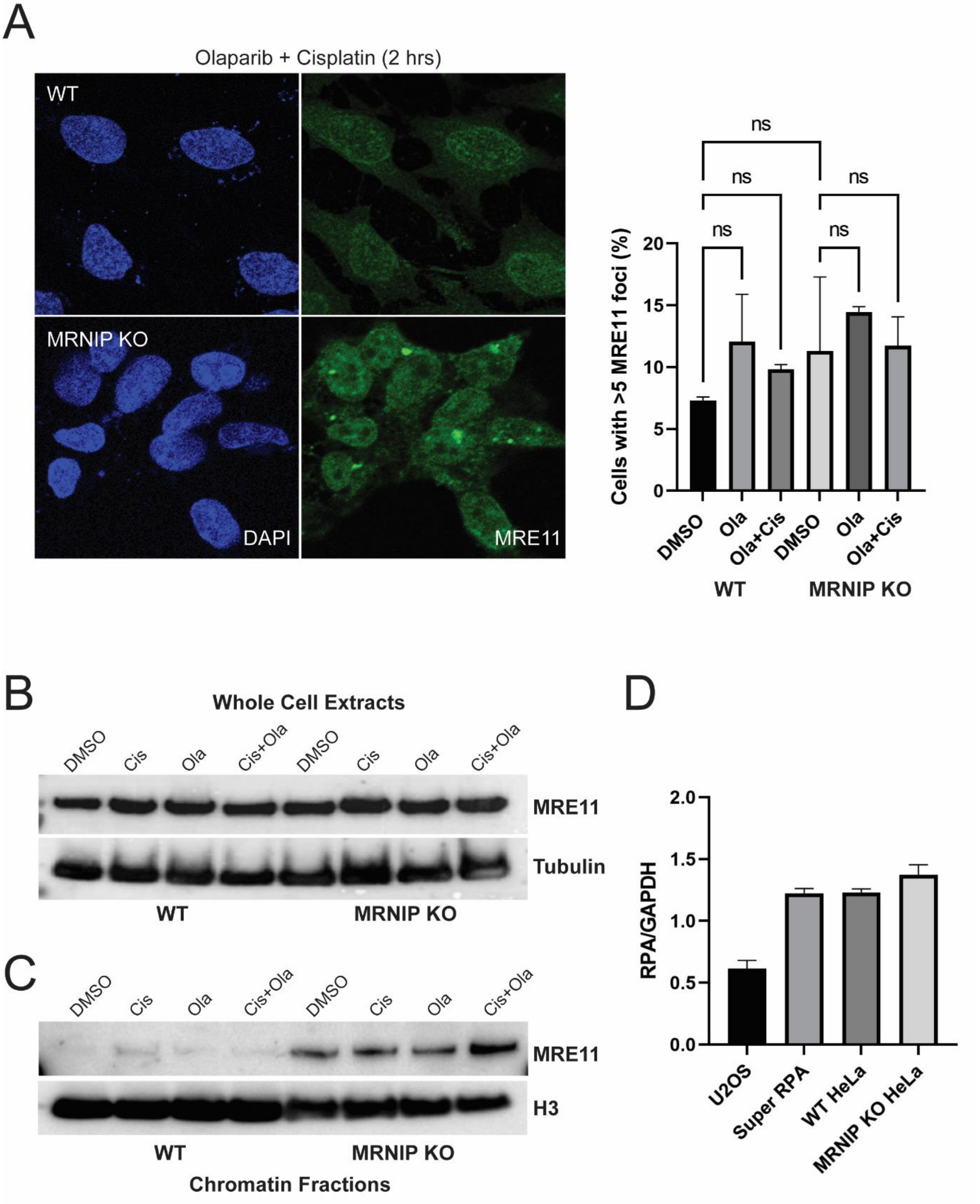

Bennett *et al.*, 2024 Supplementary Figure 2

Supplementary Figure 2: MRE11 chromatin association and localisation in MRNIP KO cells A: WT and MRNIP KO cells were treated with 1 μM Olaparib and 150 μM cisplatin for 2 hrs. The cytoplasm was pre-extracted with 0.5% Triton-X100, then cells were fixed and stained with an MRE11 antibody, and counterstained with DAPI. Representative images and quantification of the proportion of MRE11 foci-positive cells are shown. **B and C:** WT and MRNIP KO HeLa cells were treated with 1 μM Olaparib and/or 150 μM cisplatin as indicated, and MRE11 levels were either analysed in whole cell extracts (B) or in chromatin isolates (C). **D:** Quantification of RPA2 levels in U2OS, SuperRPA U2OS and WT and MRNIP KO HeLa cells (refers to Fig 4A). Band intensity was quantified using ImageJ and normalised to GAPDH to control for alterations in loading and/or total protein. All experiments were performed three times independently and analysed via one-way ANOVA. n.s = non-significant.
